# Increasing Health Behaviors and Psychological Measures with an Adapted Version of the ACCELERATION Program

**DOI:** 10.1007/s12529-024-10279-1

**Published:** 2024-04-01

**Authors:** Juliano Schwartz, Ryan E. Rhodes, Paul Oh, Shannon S. D. Bredin, Maira B. Perotto, Alejandro Gaytán González, Darren E. R. Warburton

**Affiliations:** 1 Physical Activity Promotion and Chronic Disease Prevention Unit, University of British Columbia, Vancouver, BC Canada; 2 School of Exercise Science, Physical and Health Education, University of Victoria, Victoria, BC Canada; 3 Cardiac Rehabilitation and Prevention Program, Toronto Rehabilitation Institute, University Health Network, Toronto, ON Canada; 4 West Toronto Diabetes Education Program, LAMP Community Health Centre, Toronto, ON Canada; 5 Institute of Applied Sciences for Physical Activity and Sport, University of Guadalajara, Guadalajara, Mexico

**Keywords:** Prevention, Risk factors, Chronic disease, Behavior change, Brazil, Canada

## Abstract

**Background:**

Recent evidence highlights the importance of interventions tackling physical inactivity and unhealthy eating in lower-income countries. The purpose of this study was to examine the effectiveness of the Canadian ACCELERATION lifestyle program adapted to Brazilians. The main outcomes of the study were changes in the engagement in weekly moderate-to-vigorous physical activity (MVPA) and in the daily consumption of fruits/vegetables.

**Methods:**

The adapted intervention consisted of a 12-week quasi-randomized controlled trial delivered through email. The data from the original Canadian experimental group (CE, n = 194) and the two groups of Portuguese-speaking Brazilians living in Canada in the adapted program – Brazilian experimental (BE, n = 41) and Brazilian control (BC, n = 35) – were assessed at baseline and post-intervention. The data of the 270 participants were analyzed using two-way repeated measures factorial ANCOVA (group x time) for ratio variables and Chi-square and McNemar tests for the categorical variables.

**Results:**

The BE group had a significant increase in MVPA (mean difference, 95% CI: 86.3, 38.1–134.4 min/week) and fruits/vegetables intake (3.2, 1.4–5.1 servings/day) after the intervention (both p < 0.001). The proportion of participants engaging in ≥ 150 min of MVPA increased from 4.9% to 73.2%, while adoption of a healthy diet increased from 4.9% to 53.7% in the BE group (both p < 0.001). The CE group also improved on these variables (p < 0.05) with no difference vs the BE group (p > 0.05), whereas BC did not show changes (p > 0.05).

**Conclusion:**

The Brazilian version of the ACCELERATION program effectively promoted positive health behavior changes in its participants and has the potential to contribute to the fight against risk factors for chronic diseases in Brazilians.

**Supplementary Information:**

The online version contains supplementary material available at 10.1007/s12529-024-10279-1.

## Introduction

Chronic medical conditions, including type 2 diabetes, cancer, and cardiovascular and respiratory diseases, are the leading cause of mortality worldwide [1]. These diseases share common behavioral risk factors, such as physical inactivity, low-quality diets, smoking, and excessive alcohol intake [2]. These behavioral risks are increasingly prevalent in contemporary societies due to social, political, and economic changes that have all contributed to unhealthy lifestyles [3].

The behavioral risk factors for chronic medical conditions are preventable and therefore have been receiving the attention of governments and other organizations worldwide [4–6]. Physical inactivity and the consumption of low-quality diets have increased globally and have been associated with the growing prevalence of chronic medical conditions [7–9]. According to the latest estimates, 1.4 billion adults are not physically active enough to protect their health [10], and this trend is increasing [11, 12]. At the same time, the sales of highly processed foods are soaring worldwide, with some countries consuming more than 50% of daily calories from these food products [13]. Overall, 6% of the global burden from coronary heart disease, 7% of type 2 diabetes, 10% of colon cancer, and 10% of breast cancer are due to physical inactivity [14]. Also, a higher intake of ultra-processed foods increases the risk of hypertension by 23% and is associated with a 29% higher risk of incidence of cardiovascular disease and mortality as well as a 34% higher risk of incidence of cerebrovascular disease and mortality [14].

Whereas chronic medical conditions used to be a problem chiefly observed in high-income countries, these diseases now affect low- and middle-income countries (LMICs) in a much higher proportion, with the majority of deaths due to these diseases occurring in lower-income nations [15]. Therefore, preventive initiatives centered on behavioral risk factors for chronic diseases, especially physical inactivity and unhealthy eating, are of critical, contemporary importance [16]. Such initiatives should also consider other behaviors, which despite some progress, still present global prevalence above desirable levels, particularly in terms of tobacco use [1, 17]. However, most of the evidence on how to successfully promote and facilitate physical activity and healthy eating comes from research conducted in high-income countries [16, 18]. Indeed, a variety of interventions have shown a reduction in the prevalence of chronic medical conditions in these countries [3, 19]. Examples include the National Diabetes Prevention Program and the Active People, Healthy Nation program, both from the Center for Disease Control and Prevention in the United States [20], the Move It AUS Program in Australia [21], the ParticipAction program and HealthLink BC (former Physical Activity Line) in Canada [22–24], as well as the Cycle to Work scheme in the United Kingdom [25].

Similarly, and more urgently, given the impact caused by these diseases in LMICs, poorer nations require initiatives that consider their specific contexts to tackle the burden caused by these chronic medical conditions [16, 26]. Accordingly, there is a call for action for interventions in LMICs, addressing the behavioral risk factors for chronic diseases [27–29].

With more than 205 million people, Brazil is a middle-income nation in which chronic diseases are the primary cause of mortality [1, 30, 31]. Approximately 63% of Brazilian adults are physically inactive (engagement in < 150 min of moderate-to-vigorous physical activity (MVPA) per week), 66% have a low-quality diet (consumption of < five servings of fruits/vegetables per day), and 9.1% smoke (tobacco use in any dose or route) [32]. Also, more than 26% of Brazilian adults have hypertension, around 8% have type 2 diabetes, and about 5% have cardiovascular diseases [32, 33]. Additionally, Brazil has one of the world’s fastest aging populations, with an estimated ratio of 153 seniors to 100 young individuals by 2040 – four times as many as in 2010, a substantial factor that contributes to a high prevalence of chronic diseases in the country [34, 35]. Although different actions have been taken to address these realities, such as the Family Health Strategy, significant challenges have been reported by health agents, such as lack of knowledge and protocols to properly promote healthy behaviors [36–39].

Another nation with a rapidly aging population is Canada, a high-income country where chronic diseases are also pressing health problems [40, 41]. Similar to what happens with Brazilians, more than 25% of adults in Canada have hypertension, around 9% have type 2 diabetes, and about 6% have cardiovascular diseases [41]. While the challenges to overcome the burden caused by chronic diseases in Canada are significant, including the high cost of health care associated with unhealthy behaviors, the country has consistently invested in a wide range of primary and secondary preventive actions [42, 43]. This includes the work developed by several provincial and national agencies and organizations, focusing on health promotion [44, 45]. Such initiatives have been associated with a slowdown in the incidence of chronic diseases in Canada, including the development of policies and programs that serve as models for other jurisdictions [43, 46].

As part of these initiatives, the Canadian Partnership Against Cancer developed the project Coalitions Linking Action and Science for Prevention, aimed at improving the health of Canadians [4, 47]. Considering the burden caused by risk factors for chronic medical conditions and the fact that most deaths caused by these diseases could be prevented, this Canadian Partnership Against Cancer’s initiative fostered alliances across provinces, connecting research, practice, and policy sectors. As a result, several pan-Canadian coalitions were formed, broadening the reach and deepening the impact of chronic disease prevention efforts in the country [43].

One such chronic disease prevention effort is the ACCELERATION program, a 12-week intervention to encourage health behavior change [47, 48]. A manuscript presenting the findings of the initiative is under submission, and preliminary results showed that by the end of the program, 47% of participants had started engaging in ≥ 150 min of MVPA/week, 67% had started eating ≥ five daily servings of fruits/vegetables, and 26% of individuals who smoked, quit [48]. This program involved hospitals, universities, sport clubs, companies, and government bodies in different provinces, from coast to coast of the country [49], and it is now being internationalized.

Although Brazil and Canada have significant differences, particularly in terms of socioeconomic factors, these countries share considerable similarities in terms of risk factors for chronic diseases [1, 37]. Indeed, there has been a movement in Brazil and Canada calling for initiatives addressing physical activity and healthy eating [22, 37, 50]. Considering these similarities in the health domain, both countries can mutually benefit from an exchange of knowledge. For instance, the newest version of Canada’s dietary guidelines, published in 2019, has incorporated a similar message to promote healthy eating presented in the pioneering evidence-based dietary guidelines for the Brazilian population, launched in 2014 [51, 52]. Similarly, Brazil can greatly benefit if the country follows the Canadian lead in prioritizing investments in physical activity interventions as well as chronic disease prevention initiatives in general, such as the ACCELERATION program.

While the importance of adopting healthy behaviors is well-known by most persons, it has been shown that simply having this knowledge is not enough to lead individuals to change behavior [53–55]. Recent evidence has demonstrated a gap in the relationship between intention and behavior, in that intention explains just a small portion of the engagement in physical activity and healthy eating [56, 57]. Indeed, despite having positive intentions to adopt healthy behaviors, many individuals do not increase their physical activity participation and healthy eating practices, thus suggesting that new approaches are required to tackle this situation [58–60].

Novel strategies have been proposed to bridge this intention-behavior gap [61]. Recent research highlights the importance of constructs such as affective judgment (i.e., how pleasure and enjoyment influence people’s choices), as well as perceived opportunity (the impact of social and physical environment, such as social support and resources), to engage in healthy behaviors [57, 62]. This approach has demonstrated great relevance in the adoption of physical activity and healthy eating [59, 60, 63–66].

The purpose of the present study was to examine the effectiveness of the Brazilian version of the ACCELERATION program, in order to make this intervention also available to the Brazilian population. While the Canadian initiative was based on theories from the social cognition tradition, such as the Theory of Planned Behavior [67], the adapted program added intervention content from the multi-process action control (M-PAC) schematic, such as self-monitoring, feedback on behavior, and habit formation [59, 62]. According to traditional behavior change theories, such as the Theory of Planned Behavior, the constructs of attitude and self-efficacy are key for impacting physical activity and healthy eating patterns [67]. In addition, the recent M-PAC schematic as well as the Reasoned Action Approach [68] address the discrepancy between affective (e.g., pleasant-unpleasant) and instrumental (e.g., useful-useless) judgements, as well as distinctions between perceived opportunity and perceived capability. According to the M-PAC, initially, reflective processes such as perceived capability (self-efficacy) and attitude in relation to a behavior lead to the formation of intention. Only then, affective judgements (such as expectation of enjoyment) and perceived opportunity (such as expectations of availability of time and access to engage in a behavior) translate intention into execution of the behavior [62]. The M-PAC approach was chosen to guide the Brazilian version of the program since the importance of specific constructs to better explain the intention-behavior gap regarding health behaviors, such as affective judgement and perceived opportunity, was formally presented in the literature after the delivery of the original Canadian program [57, 62]. The study’s primary hypothesis was that the Brazilian intervention would lead to positive changes in health behaviors. Additionally, it was hypothesized that the Brazilian experimental group would present better behavior change outcomes than the control group.

## Methods

### Participants

While the original Canadian program was a pre- vs. post-intervention, designed as within-subject evaluation, the adapted Brazilian program was a quasi-randomized controlled trial. The groups in this adapted version were the Brazilian experimental cohort (BE) and the waitlist Brazilian control cohort (BC). The results of the Brazilian groups were compared between each other as well as with the results of Canadian participants with similar demographics in the original program. These individuals formed the Canadian experimental cohort (CE) in the present study.

Individuals in the Canadian experimental cohort were recruited in workplaces, community centers, and healthcare facilities in different provinces across the country. All these individuals had risk factors for chronic diseases and participated in the program in different cities in Quebec, British Columbia, Nova Scotia, and Ontario. The entire material of the program was translated and culturally adapted into Brazilian Portuguese. This process involved a certified translator and a Brazilian Portuguese-speaking physiotherapist who is familiar with chronic disease prevention programs delivered in Brazil and Canada, and has advanced clinical exercise physiology training. This physiotherapist led the conduction of the adapted program, coordinating a team of Brazilian health professionals (dietitian, kinesiologist, psychologist, physiotherapist, and physician) who practiced their professions in Canada and assisted in different phases of the program.

The adapted version of the program was delivered to Brazilians living in Vancouver, British Columbia, Canada. Participants in both BE and BC were recruited mainly through official channels of the Brazilian consulate in Vancouver. To be accepted into the program, individuals had to be Brazilians living in Vancouver, aged 18 or older, primarily speakers of Brazilian Portuguese. Additionally, they should have at least one of the following criteria: engagement in less than 150 min of MVPA/week, consumption of < five servings of fruits/vegetables per day, and tobacco use in any amount.

Before and after the intervention, the participants in both BE and BC were assessed for different measures at the Physical Activity Promotion and Chronic Disease Prevention Unit at the University of British Columbia. After the intervention in the experimental group and the final assessment with both groups, the same intervention was delivered to the control group. The study was approved by the University of British Columbia Clinical Research Ethics Board (H17- 03564). All subjects in the present research signed a written informed consent form before enrolling.

## Intervention

The 12-week adapted intervention consisted of weekly online sessions, through educational videos and individual emails, focusing on self-management and motivation, using different strategies and behavior change techniques as presented by Rhodes [62] and Schwartz et al. [69]. Such approaches emphasize the importance of small changes, increasing self-regulation, nonconscious process, as well as fun and accessible opportunities, which were addressed through the use of different behavior change techniques and mechanisms of action, as presented in Table 1.

**Table 1:** Behavior change and mechanisms of action, as well as respective examples, used in the program

Behavior change technique	Mechanism of action	Topic (example)
-Information about health consequences -Focus on past success -Review behavior goals	-Knowledge; beliefs about consequences -Beliefs about capabilities -Goals	Motivation (Empathy and reminders of realistic goals; Listing positive expected outcomes and inquiring about benefits from the specific plan)
-Graded tasks -Restructuring the social environment -Restructuring the physical environment	-Beliefs about capabilities -Environmental context/resources -Behavioral cueing; environmental context/resources	Small changes—big outcomes (Strategies to progress safely and successfully; Benefits of small volumes of physical activity, small improvements in fruits/vegetables consumption and other dietary patterns, and small decreases in smoking)
-Prompts/cues -Remove aversive stimulus -Habit formation -Feedback on behavior	-Memory, attention, and decision processes -Environmental context/resources -Behavioral cueing -Motivation	Habit formation (Cues and prompts to elicit behavior change; Consistency of practices; Eating well on a budget; Provision of positive feedback and encouragement)
-Relapse prevention -Review behavior goals -Problem solving/coping planning	-Beliefs about capabilities -Goals -Beliefs about capabilities	Avoiding relapse (Identifying and planning to overcome potential challenges to translate intentions into actions; Revising goals and plans; Assistance on problem-solving)
-Instruction on how to perform a behavior -Information about emotional consequences -Restructuring the social environment -Restructuring the physical environment	-Knowledge; skills -Beliefs about consequences -Environmental context/resources -Behavioral cueing; environmental context/resources	Enjoyable physical activity (Guidance on accessing and engaging in fun physical activity; Traditional and less conventional formats and environments)
-Review behavior goals -Feedback on behavior	-Goals -Motivation	Halfway assessment (Guidance on how to do a simple and effective health and fitness assessment; Provision of positive feedback and encouragement)
-Instruction on how to perform a behavior -Information about emotional consequences	-Knowledge; skills -Beliefs about consequences	Easy healthy and happy eating (Tasty, practical, and inexpensive meal and recipe suggestions; Seasonal food availability)
-Self-monitoring of behavior -Review behavior goals -Problem solving/coping planning	-Behavioral regulation -Goals -Beliefs about capabilities	Self- regulation (Self-monitoring – reinforcement on record keeping of daily steps/MVPA, fruits/vegetables intake, reduction in smoking; Revising goals and plans; Assistance on problem-solving)
-Action planning -Feedback on behavior	-Behavioral cueing; behavioral regulation -Motivation	Time management (Adjusting priorities; Overcoming barriers to eat more produce; Provision of positive feedback and encouragement)
-Social support -Restructuring the social environment	-Social influences; environmental context/resources -Environmental context/resources	Social support beyond the program (Guidance on proactivity to identifying and establishing a social net of support, and increasing confidence)
-Information about health consequences -Salience of consequences -Restructuring the physical environment	-Knowledge; beliefs about consequences -Beliefs about consequences -Behavioral cueing; environmental context/resources	Sit less and move more (Explaining why prolonged sitting can be bad for health; How to turn sedentary activities into light physical activities)
-Review behavior goals -Feedback on behavior -Problem solving/coping planning	-Goals -Motivation -Beliefs about capabilities	Onward and upward (Reviewing key aspects of the program)

Smoking was not a concern for the vast majority of the participants. Therefore, the counselling and support related to this unhealthy behavior were provided separately through individual online interactions with the physiotherapist in charge of the intervention rather than in the educational sessions delivered to all participants.

Every week, participants were expected to spend nine minutes watching the videos and reading the emails. To check the participants’ understanding of the week’s topic and to verify adherence to the program, they were asked to answer one content-related question every week. When necessary, clarification and/or additional support was provided to assist in the intake of the content.

## Measures

The intervention’s primary outcome was a change in physical activity behavior (particularly the proportion of participants engaging in ≥ 150 min of MVPA per week). The secondary outcomes were changes in the other behaviors: healthy diet (particularly the proportion of participants eating ≥ 5 servings of fruits/vegetables per day) and smoking (proportion of smokers). The tertiary outcomes included attitude (the importance rated by participants to engage in healthy behaviors), and self-efficacy (the participants’ confidence to do so), as well as affective judgement (enjoyment related to the adoption of a behavior) and perceived opportunity (time and access to adopt a behavior). The latter two psychological measures were addressed only in the Brazilian version of the program. A final variable, the participants’ satisfaction with the program, was also part of the tertiary outcomes.

To ensure data quality, the senior researchers who contributed to the development of the Brazilian program agreed with the research coordinator to remove three questionnaires used in the original program to prevent questionnaire fatigue in the adapted version – such a decision was based on feedback from the participants in the original program. Additionally, data screening was conducted to identify participants who were not completely engaged in answering the questionnaires before and after the intervention – i.e., taking below 50% of the minimum estimated time (20 min) to complete the documents [70]. Such participants were encouraged to proceed with the total completion of the questionnaires, and two of them, who declined to do so, decided not to continue in the study, and their data was not included in the analysis (Appendix Fig. 10).

### Primary Outcome – Physical Activity

A modified version of the Godin-Shephard Leisure-Time Exercise Questionnaire was used to assess the average self-reported amount of time spent in light, moderate, and vigorous physical activity per week [71, 72] (Appendix Fig. 1). After answering the number of times engaging in each intensity category (i. e, the frequency of the activity), the participants specified the average duration of each activity. The amount of time spent in each intensity was obtained by multiplying the weekly frequency by the average of minutes engaging in light, moderate, and vigorous activities. The quantity of MVPA was determined by adding moderate and vigorous intensities together. Participants were then classified as meeting or not achieving 150 min of MVPA per week.

**Fig. 1:**
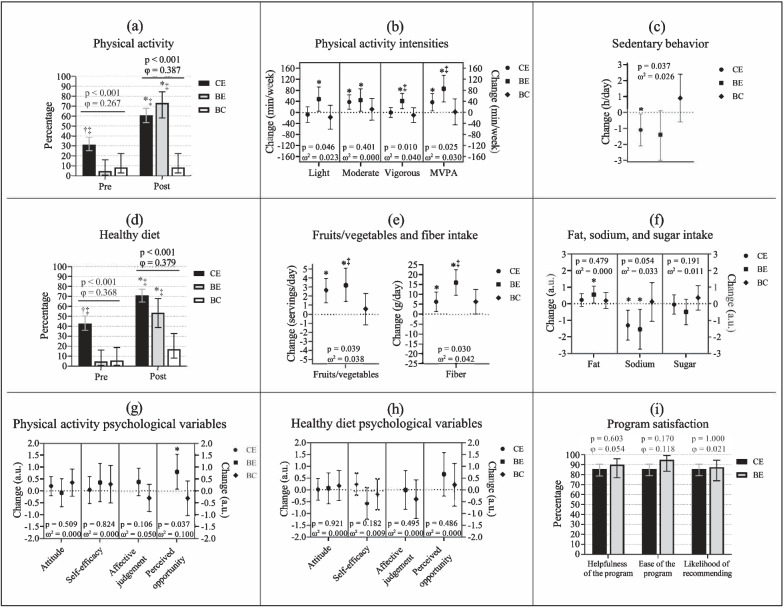
Changes in physical activity (panels *a* to *c*), diet (panels *d* to *f*), and psychological variables (panels *g* and *h*); as well as program satisfaction variables (panel *i*) compared among or between groups. Participants engaging in at least 150 min of MVPA/week compared among groups over time (**a**), changes over time in physical activity at different intensities (**b**), and sedentary behavior (**c**). Participants eating at least five servings/day of fruit/vegetables compared among groups over time (**d**), changes over time in fruits/vegetables and fiber intake (**e**), changes over time in fat, sodium, and sugar intake (**f**). Changes over time in psychological variables related to becoming or staying active for at least 150 min per week (**g**) and starting or continuing to eat five or more fruits/vegetables per day (**h**). Program satisfaction indicating the participants that rated the program ≥ 6 on a scale from 0 to 10 (**i**). Bars represent the percentage of participants per category; whiskers denote 95% confidence intervals; p-values (chi-squared) and φ statistic are for comparisons among or between groups in the same time point (panels *a*, *d*, *i*). Data presented as least squares mean and confidence intervals of 95%; p-values determined with one-way ANCOVA for comparisons among or between groups (adjusted for sex, age, marital and employment status, as well as income); omega squared (ω^2^) as effect size (panels *b*, *c*, *e*, *f*, *g*, and *h*). ^†^ Indicates a significant difference versus BE (p < 0.05); ^‡^ indicates a significant difference versus BC (p < 0.05); ^*^ indicates a significant change over time (p < 0.05). CE: Canadian experimental group (circles); BE: Brazilian experimental group (squares); BC: Brazilian control group (diamonds); MVPA: Moderate-to-vigorous physical activity

Sedentary behavior was reported through the Sedentary Behavior Questionnaire, which assessed time spent in different activities on weekdays and weekend days [73] (Appendix Fig. 2). The answers from the week were multiplied by five, and the answers from the weekend were multiplied by two, then the total was divided by seven, thereby calculating the average of sedentary time spent in hours per day.

### Secondary Outcomes – Dietary Patterns

A series of questionnaires, written specifically for this study, were presented to the participants in order to address their dietary patterns [74, 75] (Appendix Figs. 3–5). The first questionnaire had questions related to the intake of fibre, including fruits and vegetables. The second document enquired about the consumption of fat, such as milk products (cream) and full-fat salad dressing. The next document asked about sugar intake, including sweetened beverages and sweetened sauces. The final questionnaire enquired about sodium intake, which included canned and deli meat and the use of salt at the table. The answers to these questionnaires were used to determine the number of fruits/vegetables eaten per day, the total dietary fibre consumed per day, and specific scores for the consumption of fat, sodium, and sugar.

### Secondary Outcomes – Smoking

A modified version of the smoking questionnaire from the Canadian Health Measures Survey was used to assess smoking behavior [76] (Appendix Fig. 6). Participants were asked whether they smoked or not, and those who smoked were asked to answer additional questions regarding this behavior.

### Tertiary Outcomes – Psychological Measures

The initial tertiary outcomes addressed psychological aspects regarding each behavior on a 0 to 10 scale. The first question assessed attitude, and the second question assessed self-efficacy [77, 78]. The next item assessed affective judgment and the final item assessed perceived opportunity [62, 79] (Appendix Figs. 7–9). Participants were asked to circle the number that best applied to their situation about each behavior in relation to attitude, self-efficacy, affective judgement, and perceived opportunity. As previously mentioned, the last two items were presented only to the Brazilian groups.

### Tertiary Outcomes – Program Satisfaction

At the end of the intervention, program satisfaction was assessed along with the final survey. Participants were asked the following three questions:

- On a scale of 0‐10, how helpful was the program for you?
- On a scale of 0‐10, how easy was the program for you?
- On a scale of 0‐10, how likely are you to recommend this program to others?

### Statistical Analysis

Change in physical activity, particularly in the proportion of participants engaging in ≥ 150 min MVPA/week, was the primary outcome variable for statistical planning purposes. The Canadian program intended to have twice as many participants (compared to national statistics) reaching at least 150 min of weekly MVPA. Canadian statistics indicated that 15% of adult Canadians met these guidelines [80]; therefore, the goal was to have 30% of participants engaged in that amount of MVPA by the end of the intervention. Power was set at 0.80, with a 1-sided t-test to compare binomial proportions, and an alpha of 0.05 suggested a minimum sample size of 95 participants. Taking into account that 35% of adult Brazilians have been shown to meet this threshold when the intervention was designed [81], a minimum of 23 individuals was required for each Brazilian group.

Initially, data were evaluated to check accuracy and detect potential missing data. Little’s test indicated that missing data were completely at random. No more than 5% of data were missing and were handled with the expectation–maximization algorithm [82, 83]. To analyze the effects of group and time over the outcome ratio variables, we used the two-way repeated measures ANCOVA (group x time, 3 × 2; except for the affective judgement and perceived opportunity variables where the analysis was 2 × 2) adjusted for sex, age, marital status, employment, and income. We also analyzed whether the pre – post differences in the outcome ratio variables were different among groups by using the one-way ANCOVA adjusted for the same confounding variables mentioned above. The results were reported as least squares mean ± standard error of the mean (SEM), whereas the pre – post differences were reported as least squares mean and confidence intervals of 95%.

For the categorical variables, we expressed them in frequency counts and percentage, and we made comparisons among groups in the same time point by using the chi-squared test of independence and multiple z-test for proportions (Bonferroni adjusted) as post hoc. To analyze the pre – post change in the categorical variables, we used the McNemar test.

To estimate the effect size for the analyses, we calculated omega squared (ω^2^) for one-way ANCOVA comparing the pre – post differences among groups, Cohen’s d for the pre – post changes in the ratio variables, and phi (φ) and Cramer’s V for the categorical variables. The effect size was considered small, medium, or large according to the following cut-off points: 0.01, 0.06, and 0.14 for ω^2^; 0.2, 0.5, and 0.8 for Cohen’s d; and, 0.1, 0.3, and 0.5 for φ and Cramer’s V [84]. Any effect size below the small cut-off point was considered trivial [85].

Missing data for the employment variable in the CE group were imputed, since it showed the largest proportion of missing cases, thus, impeding us from having a sample size above the minimum of a priori calculated sample size (n = 95, as presented in Schwartz et al. [86]). To do so, we ran five imputations using the multiple imputation method (fully conditional specification, Markov chain Monte Carlo (MCMC)), including age and income as predictive variables.

Considering the low prevalence of smoking in the participants of the present study and the fact that nearly all assessed items related to this behavior were constant rather than variable, the results pertaining to smoking were presented descriptively.

All analyses were considered statistically significant at an alpha level of 0.05. Data were analyzed using SPSS v.27, and the graphs were drawn in GraphPad Prism v.7.04.

## Results

One hundred and twenty-five Brazilians living in Vancouver contacted the research team, interested in taking part in the adapted version of the ACCELERATION program. Of these, 41 were excluded (changed their mind or could not attend the assessments). Of the remaining 84 individuals, who were living in Vancouver for an average of 11 months, 46 were allocated to the BE group, and 38 were allocated to the BC group. Two hundred and thirty Canadians with similar demographics to the Brazilian participants were interested in taking part in the original program, forming the CE group. All three groups had participants who withdrew from the study (n = 5 in BE, n = 3 in BC, and n = 36 in CE), because of changes in their work schedule and study load, as well as pregnancy (Appendix Fig. 10). A total of 194 participants in CE, 41 in BE and 35 and BC completed the study. Given the significantly small number of Brazilians living in the Greater Vancouver Area (0.1% of the population) [87], both BE and BC had fewer participants than CE. Since it was not possible to anticipate the number of Brazilian individuals living in Vancouver who would meet all the inclusion criteria, the first participants were allocated to the BE group and those who enrolled after the establishment of the experimental group were enrolled in the BC group.

Before and after the program, for personal reasons, not all participants answered the questionnaires in their entirety. Consequently, the assessed variables for each group have different sample sizes.

Regarding the adherence to the Brazilian version of the program, 87.8% of participants answered all 12 weekly questions sent over email, 7.3% answered 11 questions, and 4.9% answered 10 questions. Also, according to self-report, 90.2% of participants spent an average of 8.5–9.0 min watching the videos and reading the emails every week, and 9.8% spent an average of 7.5–8.0 min going over the weekly material of the program.

### Baseline Descriptives

The demographic characteristics of each group (age, sex, marital and employment status, as well as income) are presented in Table 2.

**Table 2:** Baseline descriptives compared among groups. Age expressed as mean ± standard deviation, and remaining data expressed as frequency counts (percentage)

	***n*** ^**a**^	**CE**	**BE**	**BC**	***p*-Value**	**Effect Size**
Sex	194/41/35				0.001^b^	0.230^d^
Female		168 (86.6) ^g^	26 (63.4)	25 (71.4)		
Male		26 (13.4) ^g^	15 (36.6)	10 (28.6)		
Age (years)	194/41/35	47.5 ± 9.3 ^g, h^	34.9 ± 6.4	36.2 ± 6.9	< 0.001^c^	0.279^e^
Marital status	189/41/35				0.012^b^	0.182^d^
Married		140 (74.1) ^g^	39 (95.1)	28 (80.0)		
Not married		49 (25.9) ^g^	2 (4.9)	7 (20.0)		
Employment	128/41/35				< 0.001^b^	0.253^f^
Full-time		103 (80.5) ^g, h^	23 (56.1)	14 (40.0)		
Part-time		10 (7.8) ^h^	5 (12.2)	9 (25.7)		
Unemployed		15 (11.7) ^g,h^	13 (31.7)	12 (34.3)		
Income	176/40/34				0.001^b^	0.209^f^
< CAD$ 50,000/year		32 (18.2) ^g, h^	15 (37.5)	16 (47.1)		
CAD$ 50,000 to 74,999/year		36 (20.4)	10 (25.0)	7 (20.6)		
CAD$ 75,000 to 99,999/year		39 (22.2)	7 (17.5)	7 (20.6)		
> CAD$ 100,000/year		69 (39.2) ^h^	8 (20.0)	4 (11.8)		

*CE* Canadian experimental group, *BE* Brazilian experimental group, *BC *Brazilian control group, *CAD$* Canadian dollar

^a^Sample sizes for CE/BE/BC, for each variable, respectively

^b^determined with X^2^ test of independence

^c^determined with one-way ANOVA with Welch’s correction

^d^calculated as phi (ϕ)

^e^calculated as omega squared (ω^2^)

^f^calculated as Cramer’s V.

^g^Indicates a significant difference from BE within categories (p < 0.05)

^h^indicates a significant difference from BC within categories (p < 0.05)

All variables had significant differences between the Canadian and the Brazilian groups. Both BE and BC were younger than CE. The highest proportion of female participants was found in CE, with a significant difference from BE but no BC. The lowest proportion of married participants was also found in CE, which was significantly lower than BE but not BC. The CE group had the highest proportion of participants earning > CAD$ 100,000 per year, with a significant difference from BC but not BE. In contrast, CE had a lower proportion of participants earning < CAD$ 50,000 per year, which was significantly different than both BE and BC. In comparison to BE and BC, the CE group also had more participants employed full-time, and fewer unemployed participants.

### Primary Outcome—Self-Reported Physical Activity Patterns

The proportions of participants engaged in at least 150 min of MVPA/week before and after the intervention are shown in Fig. 1a. The amount of time spent in different intensities of self-reported physical activity as well as in sedentary behavior at both baseline and post-assessments are presented in Table 3. The differences in these variables over time are shown in Fig. 1b and c.

**Table 3:** Self-reported physical activity time spent at different intensities and sedentary behavior compared among groups over time. Data presented as least squares mean ± SEM

**Variable**	**Time point**	**CE (n = 173)**	**BE (n = 40)**	**BC (n = 34)**	**p-value**^**a**^
Light (min/week)	Pre	66.6 ± 10.5	30.0 ± 16.2	38.3 ± 15.9	0.167
	Post	59.0 ± 11.7	78.2 ± 18.1^b^	20.8 ± 17.7	0.038
Moderate (min/week)	Pre	50.3 ± 9.9	45.0 ± 15.3	44.8 ± 15.0	0.948
	Post	88.0 ± 10.3	89.9 ± 15.8	56.7 ± 15.5	0.167
Vigorous (min/week)	Pre	34.8 ± 7.8	12.9 ± 12.0	25.9 ± 11.8	0.349
	Post	34.2 ± 7.6	54.2 ± 11.7^b^	15.9 ± 11.5	0.039
MVPA (min/week)	Pre	85.1 ± 13.0	57.9 ± 20.0	70.7 ± 19.6	0.558
	Post	122.3 ± 15.5	144.2 ± 19.3^b^	72.6 ± 18.9	0.011
Sedentary (h/day)	Pre	7.5 ± 0.5	9.0 ± 0.8	8.5 ± 0.8	0.370
	Post	6.5 ± 0.6^b^	7.6 ± 0.9	9.4 ± 0.9	0.032

*CE* Canadian experimental group, *BE* Brazilian experimental group, *BC* Brazilian control group, *MVPA* moderate-to-vigorous physical activity

^a^determined with one-way ANCOVA for comparisons among groups in the same time point (adjusted for sex, age, marital and employment status, as well as income)

^b^Indicates a significant difference from BC within a time point (p < 0.05)

As shown in Fig. 1a, at baseline, the CE group showed a significantly higher proportion of participants engaging in at least 150 min of MVPA/week (31.5%) in comparison with the BE group (4.9%) and the BC group (8.6%). After the intervention, CE and BE showed a significant increase in the proportion of participants engaging in at least 150 min of MVPA/week (both p < 0.001), whereas no significant change was observed in BC (p = 0.999). This increase in CE (60.8%) and BE (73.2%) led to significant differences versus BC (8.6%) after the intervention.

According to self-report, the time spent in light-intensity physical activity was not significantly different among groups at baseline, whereas after the intervention, BE spent significantly more time at this intensity than BC. There was no difference among groups for vigorous physical activity before the intervention, but after the 12 weeks, the BE group had spent significantly more time at this intensity than BC. At baseline, the groups did not show a significant difference in the time spent in MVPA, whereas after the intervention BE presented significantly more minutes per week in moderate-to-vigorous intensity than BC. In terms of sedentary time, the groups spent a similar amount of time in these activities at baseline; however, the CE group showed a statistically significantly lower sedentary time than the BC group at the end of the intervention. No significant differences among groups at either time were observed for moderate intensity physical activity.

As shown in Table 3, Fig. 1b, c, participants in the BE group had significant changes in all four intensities of self-reported physical activity: 48.2 min/week in light (p = 0.032, d = 0.342), 44.9 min/week in moderate (p = 0.030, d = 0.346), 41.3 min/week in vigorous (p = 0.003, d = 0.469), and 86.3 min/week in MVPA (p < 0.001, d = 0.559), but not in sedentary behavior (-1.4 h/day, p = 0.069, d = -0.288). The CE group had a significant change in moderate (37.7 min/week, p = 0.005, d = 0.216) and MVPA (37.2 min/week, p = 0.020, d = 0.179) intensities as well as in sedentary behavior (-1.0 h/day, p = 0.036, d = -0.161) but not in light (-7.6 min/week, p = 0.599, d = -0.040) and vigorous (-0.6 min/week, p = 0.946, d = -0.005) intensities. The BC group showed no significant differences at all (light = -17.5 min/week, p = 0.423, d = -0.138; moderate = 11.9 min/week, p = 0.558, d = 0.101; vigorous = -10.0 min/week, p = 0.468, d = -0.125; MVPA = -1.9 min/week, p = 0.937, d = -0.014, and sedentary behavior = 0.9 h/day, p = 0.234, d = 0.205). The group comparison showed that BE was significantly different than BC in vigorous and MVPA intensities.

### Secondary Outcomes—Dietary Patterns

The proportions of participants reporting a healthy diet (≥ five daily servings of fruits/vegetables) at pre- and post-assessments are shown in Fig. 1d. The number of fruits/vegetables consumed per day, as well as specific scores for the intake of fiber, fat, sodium, and sugar, before and after the intervention, are presented in Table 4. The changes over time in these five variables are shown in Fig. 1e and f.

**Table 4:** Nutritional variables compared among groups over time. Data presented as least squares mean ± SEM

**Variable**	**n**^**a**^	**Time point**	**CE**	BE	**BC**	p-value^b^
Fruits/vegetables (servings/day)	106/40/34	Pre	8.0 ± 0.7^c, d^	2.3 ± 1.0	2.8 ± 0.9	< 0.001
		Post	10.7 ± 0.7^c, d^	5.5 ± 0.9	3.4 ± 0.9	< 0.001
Fibre (g/day)	106/40/34	Pre	15.3 ± 2.5^c, d^	29.3 ± 3.3	36.9 ± 3.2	< 0.001
		Post	21.5 ± 2.5^c, d^	45.2 ± 3.3	43.1 ± 3.2	< 0.001
Fat (a.u.)	105/40/34	Pre	1.9 ± 0.2	1.7 ± 0.3	1.6 ± 0.2	0.619
		Post	2.2 ± 0.2	2.2 ± 0.3	1.8 ± 0.3	0.386
Sodium (a.u.)	106/40/33	Pre	9.9 ± 0.6	8.4 ± 0.8	9.4 ± 0.8	0.336
		Post	8.6 ± 0.5	6.9 ± 0.7^d^	9.6 ± 0.7	0.008
Sugar (a.u.)	105/40/34	Pre	2.7 ± 0.3	3.0 ± 0.3	2.8 ± 0.3	0.824
		Post	2.6 ± 0.3	2.5 ± 0.4	3.2 ± 0.4	0.295

*CE* Canadian experimental group, *BE*: Brazilian experimental group, *BC* Brazilian control group

^a^Sample sizes for CE/BE/BC, respectively, for each variable

^b^determined with one-way ANCOVA for comparisons among groups in the same time point (adjusted for sex, age, marital and employment status, as well as income)

^c^ Indicates a significant difference from BE within a time point (p < 0.05)

^d^ indicates a significant difference from BC within a time point (p < 0.05)

According to Fig. 1d, at baseline, the CE group showed a significantly higher proportion of participants having a healthy diet (at least five servings of fruits/vegetables per day) (42.9%) in comparison with the BE group (4.9%) and the BC group (5.7%). After the intervention, CE and BE showed a significant increase in participants with a healthy diet (both p < 0.001), whereas no significant change was observed in BC (p = 0.125). This increase in CE (71.2%) and BE (53.7%) led to significant differences versus BC (17.1%) after the intervention.

At baseline, CE presented a significantly higher consumption of fruits/vegetables per day than BE and BC; this pattern was maintained after the intervention. Both before and after the 12 weeks, CE had a significantly lower consumption of grams of fibre per day than the Brazilian groups. Whereas at baseline there was no significant difference in sodium consumption among groups, after the intervention BE presented a lower sodium score than BC. No significant differences were found among groups for fat and sugar intake at either time point.

As shown in Table 4 and Fig. 1e, participants in the CE group showed significant increases over time in the intake of both fruits/vegetables (2.7 servings/day, p < 0.001, d = 0.376) and fibre (6.2 g/day, p = 0.014, d = 0.243). Participants in BE also showed significant increases over time in fruits/vegetables (3.2 servings/day, p < 0.001, d = 0.557) and fibre consumption (15.9 g/day, p < 0.001, d = 0.762). The BC group showed no significant change over time (fruits/vegetables = 0.6 servings/day, p = 0.515, d = 0.111; fibre = 6.2 g/day, p = 0.053, d = 0.335). The group comparison showed that BE was significantly different than BC in fruits/vegetables consumption and fibre intake.

According to Table 4 and Fig. 1f, the BE group had a significant increase over time in the intake of fat (0.5, p = 0.036, d = 0.334), as well as significant decreases in sodium (-1.5, p = 0.012, d = -0.402), but no change in sugar (-0.5, p = 0.208, d = -0.199). The CE group also showed a significant decrease in sodium (-1.3, p = 0.005, d = -0.277), but there was no significant change in fat (0.3, p = 0.242, d = 0.118) and sugar (-0.1, p = 0.878, d = -0.017). Again, BC showed no significant change over time (fat = 0.2, p = 0.435, d = 0.130; sodium = 0.2, p = 0.844, d = 0.035, and sugar = 0.4, p = 0.343, d = 0.167). The group comparison showed no significant differences in any variable.

### Secondary Outcomes—Smoking

At baseline, the CE group (n = 183) reported 11 participants to be smoking (6.0%), whereas in the BE group (n = 41) only one participant reported to be smoking (2.4%) and no participant in the BC group (n = 35) reported to be smoking. After 12 weeks of intervention, CE reported five participants that had quit smoking (2.7%), but one (0.5%) that was initially not a smoker started to smoke, leading to six participants (3.8%) smoking after the intervention, whereas in BE the single smoker had quit smoking. No changes were observed in BC.

### Tertiary Outcomes—Psychological Measures

The importance rated by participants (attitude) to engage in ≥ 150 min of MVPA/week and to eat ≥ five servings of fruits/vegetables per day, as well as their confidence to do so (self-efficacy), at baseline and after the intervention, are presented in Table 5. This table also presents the results of the other two psychological items, which were included only in the Brazilian intervention. These two variables are how pleasant (affective judgement) participants considered that it would be to engage in ≥ 150 min of MVPA/week and eat ≥ five servings of fruits/vegetables per day, and how they perceived their opportunity to do so, also at baseline and after the intervention. The differences over time in the psychological variables for each group are presented in Fig. 1g and h.

**Table 5:** Psychological measures related to physical activity (engagement in ≥ 150 min per week) and healthy diet (consumption of ≥ five servings of fruits/vegetables per day), compared among groups over time. Data presented as least squares mean ± SEM

**Variable**	**Time point**	**CE (n = 150)**	**BE (n = 40)**	**BC (n = 34)**	**p-value**^**a**^
Attitude – physical activity (a.u.)	Pre	8.7 ± 0.2	9.1 ± 0.3	8.9 ± 0.3	0.576
	Post	8.9 ± 0.2	9.0 ± 0.3	9.3 ± 0.3	0.605
Self-efficacy – physical activity (a.u.)	Pre	7.3 ± 0.3	7.9 ± 0.4	7.9 ± 0.4	0.488
	Post	7.4 ± 0.3	8.3 ± 0.4	8.2 ± 0.4	0.166
Attitude – healthy diet (a.u.)	Pre	8.8 ± 0.2	9.0 ± 0.3	9.2 ± 0.3	0.679
	Post	8.8 ± 0.2	9.1 ± 0.3	9.3 ± 0.2	0.280
Self-efficacy – healthy diet (a.u.)	Pre	7.6 ± 0.2	8.5 ± 0.4	8.4 ± 0.4	0.142
	Post	7.9 ± 0.2	7.9 ± 0.3	8.2 ± 0.3	0.683
Affective judgement– physical activity (a.u.)	Pre		8.8 ± 0.4	8.7 ± 0.4	0.953
	Post		9.1 ± 0.3	8.4 ± 0.3	0.135
Perceived opportunity– physical activity (a.u.)	Pre		7.6 ± 0.4	9.0 ± 0.4	0.016
	Post		8.4 ± 0.3	8.7 ± 0.3	0.611
Affective judgement – healthy diet (a.u.) (a.u.)	Pre		8.6 ± 0.4	8.8 ± 0.4	0.687
	Post		8.6 ± 0.3	8.4 ± 0.3	0.683
Perceived opportunity – healthy diet (a.u.) (a.u.)	Pre		8.0 ± 0.5	8.9 ± 0.5	0.271
	Post		8.7 ± 0.2	9.1 ± 0.2	0.236

*CE* Canadian experimental group, *BE* Brazilian experimental group, *BC* Brazilian control group

^a^determined with one-way ANCOVA for comparisons among groups in the same time point (adjusted for sex, age, marital and employment status, as well as income)

According to Table 5, the answers of all groups for both attitude and self-efficacy with regards to physical activity, before and after the intervention, were close to the high end of the scale. There was no significant difference among groups at both time points. Similar to the values observed for physical activity, the answers of all groups for both attitude and self-efficacy regarding healthy eating were close to the high end of the scale, before and after the intervention. Also, like physical activity, there was no significant difference among groups at both time points.

As shown in Fig. 1g and Table 5, there was no significant change over time in any group with regards to attitude (CE = 0.2, p = 0.325, d = 0.078; BE = -0.1, p = 0.783, d = -0.042; BC = 0.4, p = 0.233, d = 0.207) and self-efficacy (CE = 0.1, p = 0.901, d = 0.011; BE = 0.4, p = 0.392, d = 0.135; BC = 0.3, p = 0.487, d = 0.120) to become or stay active for ≥ 150 min/week, and there was also no significant difference among groups in these two variables in relation to physical activity. Regarding diet, as shown in Fig. 1h, there was also no significant change over time in any group in attitude to start or continue to eat ≥ five daily servings of fruits/vegetables (CE = 0.0, p = 0.970, d = 0.004; BE = 0.1, p = 0.864, d = 0.029; BC = 0.1, p = 0.595, d = 0.088), nor in self-efficacy (CE = 0.3, p = 0.248, d = 0.092; BE = -0.6, p = 0.114, d = -0.251; BC = -0.2, p = 0.643, d = -0.083). No significant differences were found among groups for any variable.

Similar to attitude and self-efficacy, the answers related to affective judgement and perceived opportunity to become or stay active for ≥ 150 min/week as well as to eat ≥ five servings of fruits/vegetables per day in BE and BC were close to the high end of the scale (Table 5). Affective judgement did not show significant differences between groups at any time point. However, the BE group showed significantly lower perceived opportunity in relation to physical activity than the BC group at baseline, but it was no longer significantly different after the intervention.

According to Table 5 and Fig. 1g and h, there was no significant change over time in any group in affective judgement (BE = 0.3, p = 0.205, d = 0.209; BC = -0.3, p = 0.296, d = -0.184), whereas BE showed a significant change over time in perceived opportunity to become or stay active for ≥ 150 min per week (0.8, p = 0.034, d = 0.351) but BC did not (-0.3, p = 0.394, d = -0.148). Likewise, there was no significant difference between groups in affective judgement, but it did significantly differ in perceived opportunity in relation to physical activity. There was also no significant change over time in any group in affective judgement (BE = 0.0, p = 0.984, d = -0.004; BC = -0.4, p = 0.323, d = -0.171), nor perceived opportunity to start or continue to eat five or more fruits/vegetables per day (BE = 0.7, p = 0.156, d = 0.232; BC = 0.2, p = 0.645, d = 0.080). Also, there was no significant difference between groups in these two variables in relation to diet.

### Tertiary Outcomes—Program Satisfaction

Rates ≥ 6 were used to determine the participants’ satisfaction with the program. The proportions of participants who considered the intervention helpful, easy, and would recommend it are shown in Fig. 1i. There was no difference between groups in the proportions of participants who considered the program helpful, easy, and would recommend it.

## Discussion

The overall purpose of this study was to examine the effectiveness of the ACCELERATION program adapted to the Brazilian population. The BE group had significant positive changes in health behaviors and presented similar results to the CE group. These findings confirm the primary hypothesis of the study and show that this adapted program was as efficacious as the original intervention. Additionally, the Brazilian experimental group presented better behavior change outcomes than the control group, which confirms the second hypothesis of the project.

### Primary Outcome—Self-Reported Physical Activity Patterns

The number of participants in the BE group engaged in ≥ 150 min of MVPA per week drastically increased after the intervention, from 4.9% to 73.2%. The CE group also had a considerable increment in the number of participants engaging in at least 150 min of MVPA/week, from 31.5% to 60.8%. According to these results, both groups were effective in inducing an increase in physical activity rates and presented better results than the BC group, with a medium to large effect size.

The effectiveness of both interventions can be confirmed by the increases in the minutes of weekly MVPA of 149.1% in BE (medium to large effect size) and 43.7% in CE (trivial effect size), whereas BC remained the same (trivial effect size). With this substantial increase, BE promoted better results than BC. The results observed in the BE group are equivalent or better than those found in studies with similar characteristics, targeting adults at risk of chronic diseases although overall, BE had fewer participants than the sample sizes in these other studies. A meta-analysis with six randomized controlled trials assessing self-reported MVPA reported overall medium effects [88]. With an average duration between 8 and 12 weeks, these interventions included the use of phone text messages, wearable devices, phone apps, and emails.

The BE group also had an increase in the weekly minutes of light physical activity of 160.7% (small to medium effect size), a better result to induce this change than BC (trivial effect size), which had no change, as was also the case of CE (trivial effect size). In terms of sedentary behavior, only the CE group had a significant change—a decrease of 13.3%, with a trivial effect size. A possible reason for the absence of change in this behavior in the BE group is the fact that many items in the questionnaire are not mutually exclusive, which can lead individuals to report many more hours than the real time spent being sedentary [73]. This is the case, for example, of the third item (sitting while listening to music) and the last item (sitting and driving/riding in a car, bus, or train). Although this could have also happened with CE, this may not have been an issue given the larger sample size of this group [89]. The limited accuracy of the assessment instrument is acknowledged by the authors of the study on the reliability and validation of the Sedentary Behavior Questionnaire [73]. Nevertheless, according to a meta-analysis, the tool had the best performance among questionnaires evaluating this behavior when compared against device-assessed sedentary time [90].

It seems that the use of a number of components instead of only one (such as an activity tracker or email messages) to support participants during the intervention, as proposed by Smith et al. [121], corroborated the positive results of the present trial in terms of physical activity patterns. As well, the adoption of different behavior change techniques, including those focusing on enjoyment and attainability, likely contributed to these findings, which is in agreement with recent theories ([62] and Rhodes and Rebar (2018). Moreover, encouraging individuals to perform as much physical activity as they considered possible, whenever and wherever suitable for them, without demanding a minimal threshold nor requiring practices in specific locations, as it has been increasingly suggested in the last years (Segar et al., 2020; Thomas Craig et al., 2020; Warburton and Bredin 2017), probably also contributed to the results observed in this trial. Since not many interventions have applied the same behavior change techniques as the present research, future studies with similar approaches and larger samples are warranted to provide a further understanding on the relationship between these strategies and the positive results observed in this trial.

### Secondary Outcomes—Dietary Patterns

There was a substantial increase in the proportion of participants in the BE group eating ≥ five servings of fruits/vegetables per day, from 4.9% at baseline to 53.7% after the intervention. The CE group also had a considerable increment, from 42.9% to 71.2%. These results indicate that both experimental groups were effective in increasing the prevalence of healthy diets and presented better results than BC, with a medium to large effect size. Also, the increase in the number of daily servings of fruits/vegetables in the two experimental groups confirms the efficacy of both interventions. The BE group had an increment of 139.1% (medium to large effect size) and the CE group increased 29.5% (small to medium effect size), whereas the BC group remained the same (trivial effect size). This smaller increase in CE can be explained by the fact that this group already had a considerably higher consumption of fruits/vegetables at baseline, which was three and a half times higher than BE.

Studies similar to the present research, focusing on chronic disease prevention, reported less convincing results. A meta-analysis focused on technological tools, such as mobile phones and the internet, with 11 interventions aiming at increasing the consumption of fruits and vegetables, reported positive changes with a small to medium effect size [91]. These studies used a variety of behavior change techniques and had an average duration of 12.7 weeks. Fibre intake, which also included items other than fruits and vegetables, such as whole grains and beans, increased in both experimental groups, by 40.5% in CE, with a small to medium effect size, and 54.3% in BE, with a medium to large effect size. The BE group achieved better results than the BC group, which did not change (small to medium effect size). Sodium intake also improved in the experimental groups, with a decrease of 13.1% in CE and 17.9% in BE, both with small to medium effect sizes, while BC did not change (trivial effect size). No group presented any change regarding the intake of sugar.

A systematic review and meta-analysis on the effectiveness of interventions for improving dietary intake grouped the studies into those where the intervention produced a significant change and those where this did not happen [92]. Although the studies had different purposes and applied varying methodologies to collect data on different diet components, two of them were similar to the present study: an online intervention, aimed at preventing weight gain by providing educational sessions on a digital platform and feedback via email, observed no change in the fibre intake [93], and an intervention based on a smartphone app and counselling sessions with a health coach, assessed the consumption of sugar and sodium, and also did not observe changes over time [94]. Unlike the other dietary variables, BE had a worsening in the consumption of fat, with an increase of 29.4% (small to medium effect size). A probable explanation for this finding is that a change in the dietary patterns of the participants in this group, such as an increase in the consumption of fruits and vegetables, involved an adjustment/compensation in the eating behavior with a higher intake of fatty foods. Possible examples include snacks or desserts consisting of fruits with whipped cream as well as snacks or meals containing vegetables seasoned with full-fat salad dressings. Eating more fat while adjusting to a healthier diet has also been reported in other studies [95, 96].

Overall, the dietary results of the present trial were equivalent or better than those from similar studies – although it is important to keep in mind the small sample size of the Brazilian groups. Based on the findings of two recent meta-analyses focusing on healthy eating, there are two possible aspects that might help explain these findings: the duration and the number of behavior change techniques used in this intervention. Ashton et al. [92] reported that larger increases in the intake of fruits/vegetables are observed after three months of intervention when compared to interventions with longer durations. Also, according to Rodriguez Rocha and Kim [91], larger effect sizes are presented in studies applying seven or more behavior change techniques. The Brazilian version of the ACCELERATION program lasted three months and used around 20 behavior change techniques, most of which were applied in contexts related to diet [86]. Specifically regarding fat intake however, the program did not present the same positive results; indeed, this variable worsened after the intervention. Therefore, it is recommended that future studies and practical interventions addressing this variable consider alternative approaches.

### Tertiary Outcomes—Psychological Measures

In line with what was observed in similar interventions, the average findings regarding the psychological variables of the present study were close to the high end of the scale at the beginning of the program, not allowing much room for improvement [97, 98]. Indeed, despite the use of a few behavior change techniques focusing on beliefs and capabilities, no group observed any change in attitude (perceived importance) or self-efficacy (confidence) regarding physical activity and diet. According to Rhodes [62], this happens because attitude and self-efficacy can sometimes be initiating processes (i.e., not considered antecedents of the translation of an intention), and thus not as important to daily fluctuations in behavioral performance among people who already have the intention to change behavior (such as those beginning a behavior change study). Instead, affective judgement and perceived opportunity are predictors of the translation of an intention, in that they represent emotional and operational challenges that one faces in daily decisions to engage in a behavior. By presenting more variance, these constructs are considered ongoing reflective processes [99].

A meta-analysis of interventions based on smartphone apps to promote physical activity reported similar findings to the present study, i.e., an increase in physical activity and no change in self-efficacy [100]. No meta-analysis addressing diet with a focus on interventions using technology support was found. However, a meta-analytic review with diverse populations and different delivery modes did include nine studies with similar characteristics to the present study [101]. Equivalent results were found in one intervention: an internet-based study reported an increase in healthy eating with no change in self-efficacy [102], and two other interventions, also delivered via internet, observed positive outcomes in healthy eating along with a decrease in self-efficacy [103, 104]. Three other internet-based studies, however, observed an increase in both dietary patterns and self-efficacy [105–107], and Irvine et al. [106] also assessed attitude, observing the same results. Alternatively, although three other internet-based interventions also reported increases in healthy eating behavior and self-efficacy, participants in these studies have reported low scores in self-efficacy at baseline, thus allowing more room for improvement through the intervention [108–110]. Additionally, Cook et al. [108] also measured attitude, which had the same pattern. Regarding attitude towards physical activity, only one study similar to the present research, which measured this variable as well as physical activity behavior was found [111]. Such an intervention consisted of a running program delivered online, with the support of a social media network. Similar to the present study, the authors observed an increase in physical activity but not in attitude towards this variable. Moreover, a two-year phone-based trial, which investigated determinants of motivation to engage in healthy behaviors and was issued after the publication of the meta-analyses from Silva et al. [100] and Prestwich et al. [101], did not observe changes in attitude towards physical activity and healthy eating nor in self-efficacy regarding these two behaviors [98].

With regards to the other psychological measures, the BE group had an increase in affective judgement and perceived opportunity regarding physical activity, as well as perceived opportunity regarding diet. While the increase in perceived opportunity to become or stay active for ≥ 150 min/week was significant and clinically meaningful (small to medium effect size), with BE presenting better results than BC, the other variables did not reach significance. However, the increase in affective judgement to become or stay active for ≥ 150 min/week as well as the perceived opportunity to eat ≥ five daily servings of fruits/vegetables were clinically meaningful (small to medium effect size). In the BC group, all variables had a non-significant decrease, except for perceived opportunity to start or continue to eat ≥ five daily servings of fruits/vegetables, which had a non-significant and non-clinically meaningful increase.

Motivation theories have been evolving, targeting physical activity and healthy eating, which were not considered in the development processes of traditional theories addressing health behaviors [57, 62, 112]. Thus, constructs of significant importance to explain and predict physical activity and healthy eating, such as affective judgment and particularly perceived opportunity, have just recently started to receive more attention; therefore, fewer lifestyle trials have investigated these variables in comparison to other psychological measures such as instrumental attitude and particularly self-efficacy [113, 114]. In a meta-analysis focusing on affective judgement and physical activity [115], two studies were similar to the present research. A trial based on text messages reported increases in both physical activity and affective judgement [116]. Alternatively, a study comparing fitness lessons only versus fitness lessons plus access to a social media group with additional information, such as videos and articles about health and fitness, reported that both groups increased physical activity without increasing affective judgement [117].

Regarding perceived opportunity, no similar study assessing changes in this variable over time was found. However, this construct has been an important part of recent interventions addressing both physical activity and healthy eating behaviors, such as a 10-week blended nutrition program with in-person and online sessions, focusing on healthy weight [66], and an eight-week physical activity intervention, delivered through lessons using a smartphone, focusing on incentives for physical activity engagement [118].

The psychological variables’ scores of the present study demonstrate that participants in all groups were highly motivated to engage in behavior change at baseline and kept this motivation throughout the study. As such, it is not common for variables related to these constructs to present significant changes over time [98, 119, 120]. The fact that both physical activity and healthy eating had overall positive changes while most of the psychological variables did not have significant improvements may be related to two aspects observed in this trial. One reason is similar to what happened in the study conducted by Caini et al. [98]: the frequent contact from the participants with the researcher during the email exchanges addressing different aspects throughout the Brazilian version of the ACCELERATION program, which happened after the initial interactions during the recruitment period and assessments [86]. According to personal communications during and particularly after the program, participants felt cared for and constantly encouraged in their efforts. According to Young et al. [120], another probable motive is the fact that some participants reached their goals in terms of health behavior change before the end of the intervention, which would likely limit increases in the constructs related to motivation.

While it is important not to generalize the results of this trial because of the small sample sizes, the results of the study indicate that tailored interactions with participants, considering their individual characteristics and preferences, with the use of behavior change techniques emphasizing enjoyment and attainability, have the potential to elicit health behavior change. This finding is corroborated by recent studies addressing these topics, such as the systematic review and meta-analysis from Smith et al. [121], the state-of-the-art review from de Ridder et al. [57], and studies about the intention-behavior gap [59, 61, 62].

### Tertiary Outcomes—Program Satisfaction

More than 85% of participants in each experimental group considered the program helpful, easy to follow, and would recommend it. The fact that there was no difference in the levels of satisfaction between BE and CE reinforces that the Brazilian version was as effective as the Canadian program. The study’s findings regarding satisfaction are equivalent or better than those from similar studies. In a six-month text-message based intervention, focusing on behavior change strategies aiming at weight management, 70% of the participants considered the program useful, and 75% reported being satisfied with it [122]. Two other studies did not present the proportion of participants satisfied with the interventions but did report the general level of the participants’ satisfaction. To assess the effects of an app for monitoring physical activity, a 12-week intervention was conducted addressing behavioral aspects such as self-efficacy and goal setting [123]. The authors reported that, likely because of technical issues with the app, the participants provided lower rates of satisfaction than the control group, which used a commercial activity tracker only. And in an intervention using a website to provide tailored information to pursue healthy behaviors, participants also did not provide high rates of satisfaction [124]. According to the authors, this suggests that the participants found that the website was not especially helpful, and that tools with interactive features would be more beneficial.

The findings of the Brazilian version of the ACCELERATION program regarding the participants’ satisfaction demonstrate that the structure of the intervention was appropriate to please the vast majority of those registered in the study. These results indicate that this program’s design may be an effective alternative to promote health behavior change in adults at risk of chronic diseases.

## Limitations

The Brazilian version of the ACCELERATION program had a smaller sample size than the original intervention, was not a true randomized controlled trial, and the sample was not representative; therefore, it should not be generalized to the entire population of interest. However, this sample size was based on appropriate testing power, which contributed to lowering the impact related to the sample size. Additionally, although the Brazilian groups were not truly randomized, a high level of reliability of the findings can be observed, since no difference was found between these groups at baseline.

Out of the 183 individuals who answered the tobacco use questionnaire in the CE group, only 11 reported being smokers at baseline. A similar observation was recorded with just one out of 41 participants in the BE group, while no participant was smoking in the BC group. Both experimental groups were effective in reducing the number of smokers. After the program, the number of smokers decreased to five in CE, and BE had no more smokers. These small numbers of participants who smoked before the program reflect the low prevalence of smokers in these communities, a trend also observed in other lifestyle interventions conducted in the twenty-first century [124–126]. Therefore, future interventions targeting this behavior could benefit from a more specific recruitment process. Additionally, the dietary questionnaires have been presented at scientific conferences of the American Heart Association and subsequently published in Circulation (the official journal of the American Heart Association). However, these forms were not validated. While it cannot be guaranteed that the questionnaires capture the variables they aim to measure, this does not affect the comparison between groups.

Also, the main emphasis of psychological outcomes assessment of this adapted intervention was on the social cognitive “reflective” layer of M-PAC, and correspondent with the Reasoned Action Approach. However, because some of the intervention content was applied to target regulatory and reflexive processes, it would have been ideal to also explore changes to behavioral regulation, habit, and identity. Therefore, it is recommended that future interventions include these measures in the assessments. The present study also did not include a follow-up period, which could show whether the changes observed immediately following the intervention were maintained. Accordingly, a subsequent study should include a follow-up stage after the delivery of the Brazilian version of the ACCELERATION program, to determine the aspects involved in the maintenance of any changes over longer periods of time.

## Conclusions

The Brazilian adapted version of the ACCELERATION program presented similar results to the original protocol and led to improvements in health behaviors and psychological measures supporting the efficacy and effectiveness of the intervention. Also, the Brazilian experimental group presented better results than the control group in the majority of the healthy lifestyle measures. Although the number of participants in the Brazilian intervention was smaller than the Canadian one, the overall higher effect sizes of the results of the present trial in comparison to similar studies, along with the levels of satisfaction of the participants, confirm that the translated and adapted intervention was effective in promoting positive health behavior changes in Brazilians living in Vancouver. A scaled-up initiative based on this program may contribute toward the prevention of chronic diseases in this population.

## Supplementary Information

Below is the link to the electronic supplementary material.

Supplementary file1 (DOCX 1738 KB)
