# Supplementary material for: Increasing Health Behaviors and Psychological Measures with an Adapted Version of the ACCELERATION Program
Source: Int J Behav Med. 2024 Apr 1;32(2):195–213. doi: 10.1007/s12529-024-10279-1 (PMC12031918; doi:10.1007/s12529-024-10279-1)
Supplement: Supplementary file 1 — Supplementary file1 (DOCX 1738 KB) [file 12529_2024_10279_MOESM1_ESM.docx]

Figure 1. Physical activity questionnaire.

Figure 2. Sedentary behaviour questionnaire.

Figure 3. Diet questionnaires, page 1/3.

Figure 4. Diet questionnaires, page 2/3.

Figure 5. Diet questionnaires, page 3/3.

Figure 6. Smoking questionnaire.

Figure 7. Psychological measures, page 1/3.

Figure 8. Psychological measures, page 2/3.

Figure 9. Psychological measures, page 3/3.


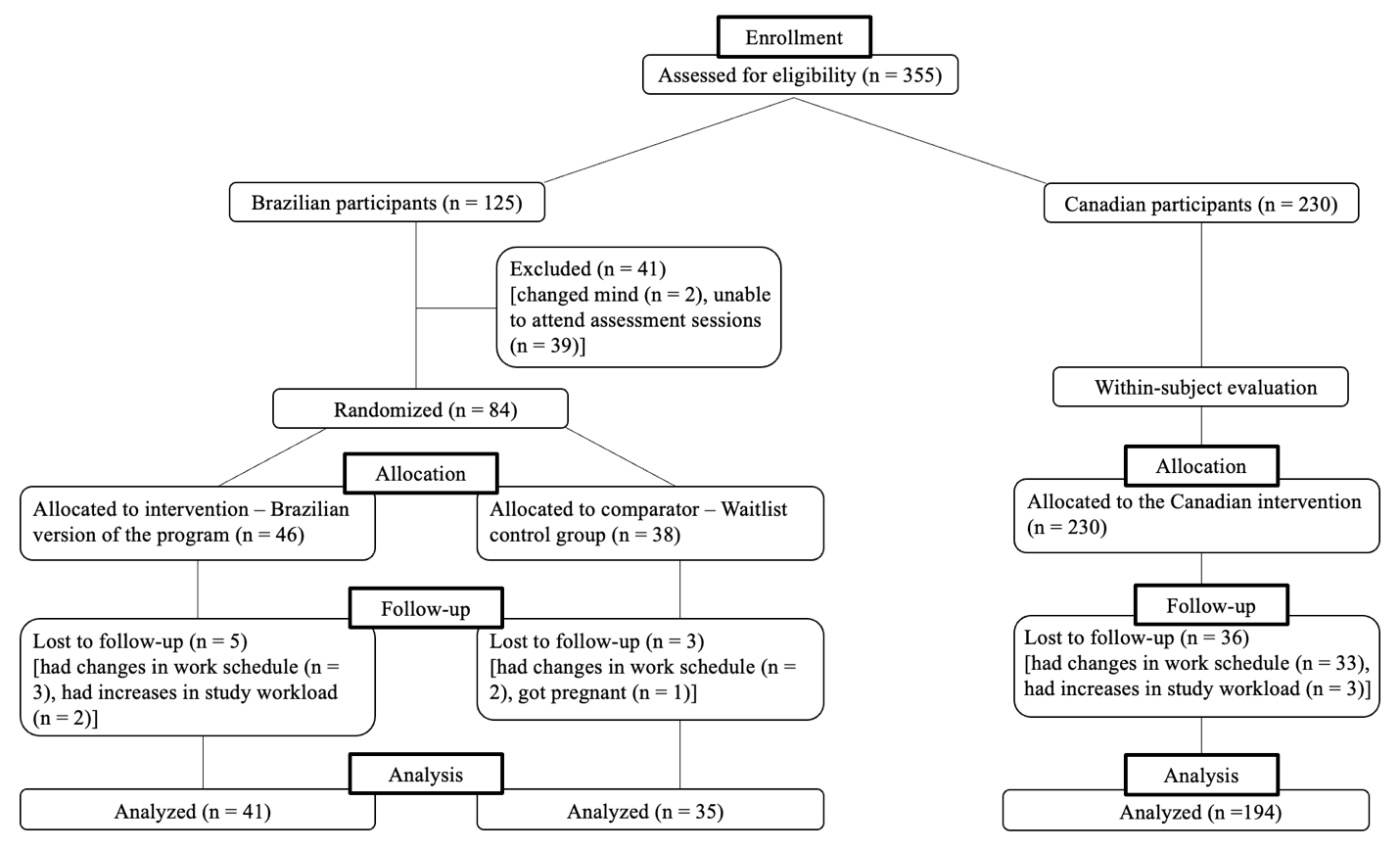
 Figure 10. Study Flow**.**
